# Supplementary figures and images for: Quantifying T Cell Cross-Reactivity: Influenza and Coronaviruses
Source: Viruses. 2021 Sep 7;13(9):1786. doi: 10.3390/v13091786 (PMC8472275; doi:10.3390/v13091786)

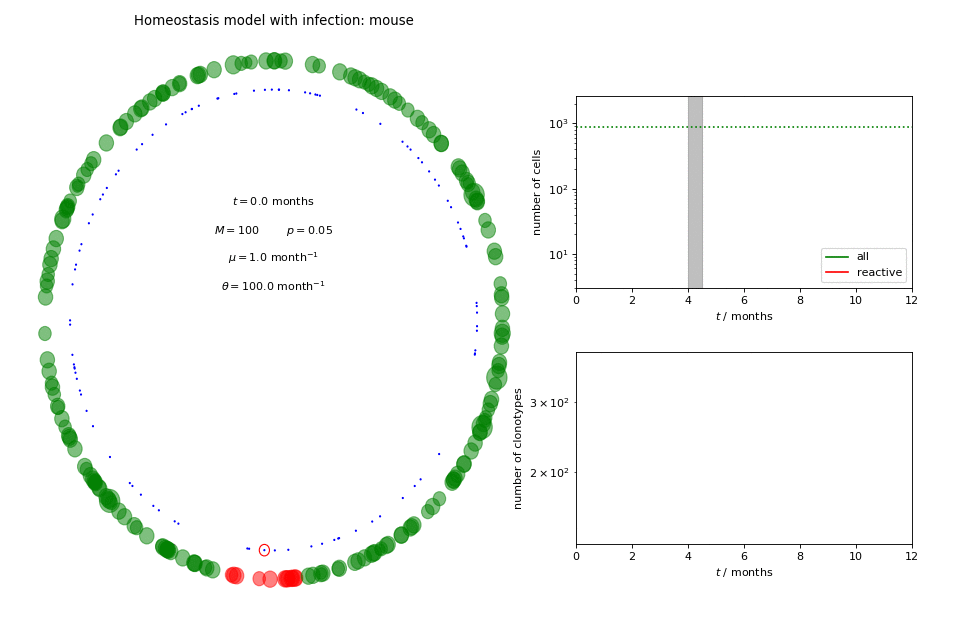

Supplement: Supplementary file 1 [file viruses-13-01786-s001.zip › RepertoireJune2021.gif]
